# Maternal Dietary Supplementation with Oligofructose-Enriched Inulin in Gestating/Lactating Rats Preserves Maternal Bone and Improves Bone Microarchitecture in Their Offspring

**DOI:** 10.1371/journal.pone.0154120

**Published:** 2016-04-26

**Authors:** Pilar Bueno-Vargas, Manuel Manzano, Javier Diaz-Castro, Inmaculada López-Aliaga, Ricardo Rueda, Jose María López-Pedrosa

**Affiliations:** 1 Abbott Nutrition R&D, Granada, Spain; 2 Department of Physiology, Faculty of Pharmacy, University of Granada and Institute of Nutrition and Food Technology “José Mataix”, University of Granada, Granada, Spain; The University of Manchester, UNITED KINGDOM

## Abstract

Nutrition during pregnancy and lactation could exert a key role not only on maternal bone, but also could influence the skeletal development of the offspring. This study was performed in rats to assess the relationship between maternal dietary intake of prebiotic oligofructose-enriched inulin and its role in bone turnover during gestation and lactation, as well as its effect on offspring peak bone mass/architecture during early adulthood. Rat dams were fed either with standard rodent diet (CC group), calcium-fortified diet (Ca group), or prebiotic oligofructose-enriched inulin supplemented diet (Pre group), during the second half of gestation and lactation. Bone mineral density (BMD) and content (BMC), as well as micro-structure of dams and offspring at different stages were analysed. Dams in the Pre group had significantly higher trabecular thickness (Tb.Th), trabecular bone volume fraction (BV/TV) and smaller specific bone surface (BS/BV) of the tibia in comparison with CC dams. The Pre group offspring during early adulthood had an increase of the lumbar vertebra BMD when compared with offspring of CC and Ca groups. The Pre group offspring also showed significant increase *versus* CC in cancellous and cortical structural parameters of the lumbar vertebra 4 such as Tb.Th, cortical BMD and decreased BS/BV. The results indicate that oligofructose-enriched inulin supplementation can be considered as a plausible nutritional option for protecting against maternal bone loss during gestation and lactation preventing bone fragility and for optimizing peak bone mass and architecture of the offspring in order to increase bone strength.

## Introduction

Osteoporosis has been recognized as an established and well-defined disease that affects millions of people around the world[1]. It is defined by the National Institute of Health as a skeletal disorder characterized by compromised bone strength that increases the risk of fracture[2]. The bone mass of an individual in adult life depends on the peak attained during skeletal growth and the subsequent rate of bone loss [3, 4]. In this sense, the quantity of bone could be affected during different periods along individual’s life. Two of the most important periods could be childhood and adolescence, being the increment of peak bone mass attained during these periods one of the most important strategies for preventing osteoporosis and associated fractures later in life. Other important periods that could affect in a significant way the bone mass and structure are gestation and lactation. During gestation and especially during lactation a high quantity of Ca is mobilized, mainly from maternal skeleton, to meet the Ca requirements for foetal and neonatal growth and milk production[5]. In the course of these periods, a rapid and consistent fall in maternal bone mass is produced in parallel with the increase in the bone turnover rate [6] Thus, it has been estimated that losses of bone mineral content (BMC) and bone mineral density (BMD) are between 5% and 14% resulting in the development of a transient maternal osteopenia that normally is recovered after weaning but, in some rare cases, could produce backache and vertebrae fractures in the lactating women [7–9].

Gestation and lactation have been also related as principal factors that could have an impact on bone accrual of the offspring during the growing stages of life, leading to an adequate peak bone mass. In recent years, environmental factors during intrauterine life, such as maternal nutrition, have been identified to influence the growth and development of children [10]. The relatively rapid rate of mineral gain during intrauterine and early postnatal life, coupled with the plasticity of skeletal development in the uterus, offer the possibility of profound interactions between the genome and the environment at this stage of life [11, 12]. This phenomenon termed as ‘programming’ was defined as “persisting changes in structure and function caused by adverse environmental influences at a critical stage of early development” [13]. Thus, maternal intake of nutrients such as proteins, fats, minerals (Ca, P, Mg, K), and vitamins (folate and vitamin D) during pregnancy has been shown to predict height, BMC, bone area, and areal bone mineral density in prepubertal children [14–17].

Ca is the most widely evaluated nutrient in prospective and interventional human studies with the aim of establishing the relationship between maternal dietary status, maternal bone health and offspring skeletal development[18, 19]. The National Academy of Sciences recommends for women who are pregnant or breastfeeding to consume 1,000 mg of Ca each day. In fact, different studies have shown that increased Ca intake during gestation and lactation can preserve maternal bone and can also promote a higher peak bone mass in the offspring [10, 16, 20]. However, other studies have not shown this positive correlation between both variables [21], leading to consider other alternative technologies by the scientific community.

Alternative, other nutrients and functional foods are being studied for their programming abilities. One of the candidates, on which it is gaining knowledge in this area, is prebiotics. A prebiotic is a non-digestible food ingredient that benefits the host by selectively stimulating the growth and/or activity of probiotic bacteria/s in the colon that could deliver potential benefits for the host health[22]. In fact, beneficial effects of prebiotic fibres are been observed on immunological, metabolic and gastrointestinal systems [23, 24].

Among the most studied prebiotics are those derived from chicory: inulin and oligofructose (FOS). Both prebiotics are natural constituents of vegetables, fruits and cereals and have been recognised as dietary fibres in most countries[25]. FOS and inulin have been associated with increases of calcium absorption in rodents[26] and in humans[27] and also with a clear beneficial effect on bone metabolism in adolescent [28, 29] and postmenopausal model[30]. Thus, long-term prebiotic supplementation in growing rats has shown to increase not only the accumulation of bone mineral but also to improve the trabecular structure of the bone[31, 32] even when rats were fed with a low calcium diet (0.2%)[33]. Similarly, prebiotics supplementation of the diet prevent trabecular bone loss in different animal models of osteoporosis using ovariectomized rats[34] and mice[35]. In humans, prebiotics consumption has been related with higher calcium absorption in the adolescence if fed short-term[36, 37] that have a significant higher impact on bone mineral content of young population if fed during a long-term period[28]. These results could indicate that prebiotic consumption during pubertal growth enhances bone mineralization that could result in an increase in the peak bone mass during adolescence[38]. Although there are promising data of the effect of the prebiotic supplementation in humans and animals, no studies were found describing the programming effects of prebiotics on maternal and offspring bones.

The aim of our study was to determine in rats the effects of maternal diet supplementation with prebiotic oligofructose-enriched inulin during gestation and lactation, on the prevention of maternal bone loss at weaning and on the offspring peak bone mass and architecture accretion. In the present study we assessed the possible effects of prebiotic oligofructose-enriched inulin on the density and microstructure of axial and appendicular bones as well as bone biomarkers to assess biological status of the rats.

## Material and Methods

### Animals and experimental design

This study was carried out in strict accordance with the recommendations in the ethical guidelines for animal experimentation provided by the Spanish National Research Council (RD 1201/2005 October 10). The protocol was approved by the Committee on the Ethics of Animal Experiments of the Unidad de Experimentación Animal-Estación Experimental del Zaidín (CSIC, Granada, Spain) (Permit Number: CEEA-INAN-2008-004). All interventions were performed under sodium pentobarbital anesthesia, and all efforts were made to minimize suffering.

Thirty pregnant Sprague-Dawley rats, 15wks old, at 11^th^ gestation day were obtained from Charles Rivers Laboratories (Orleans Cedex, France). The dam rats were housed under standardized environmental conditions (22°C, relative humidity of 50%, on a 12 h light/dark cycle). They were randomly divided into three groups according to the feeding pattern (n = 10/group): the Control group (CC group), which received purified rodent diet, AIN93-G [39]; the Ca-fortified group (Ca group), which received AIN93-G diet fortified with 0.5% calcium carbonate (CaCO_3_) i.e., 1 g Ca^2+^/100 g of diet, and the prebiotic oligofructose-enriched inulin group (Pre group), which received AIN93-G diet containing 7.5% of the total carbohydrates as prebiotic oligofructose-enriched inulin (Synergy-1®, Orafti, Belgium). This prebiotic was a 1:1 mixture of fructooligosacharides, with an average degree of polymerization (DP) of 4, and high performance inulin with an average DP of 25.

**Fig 1:**
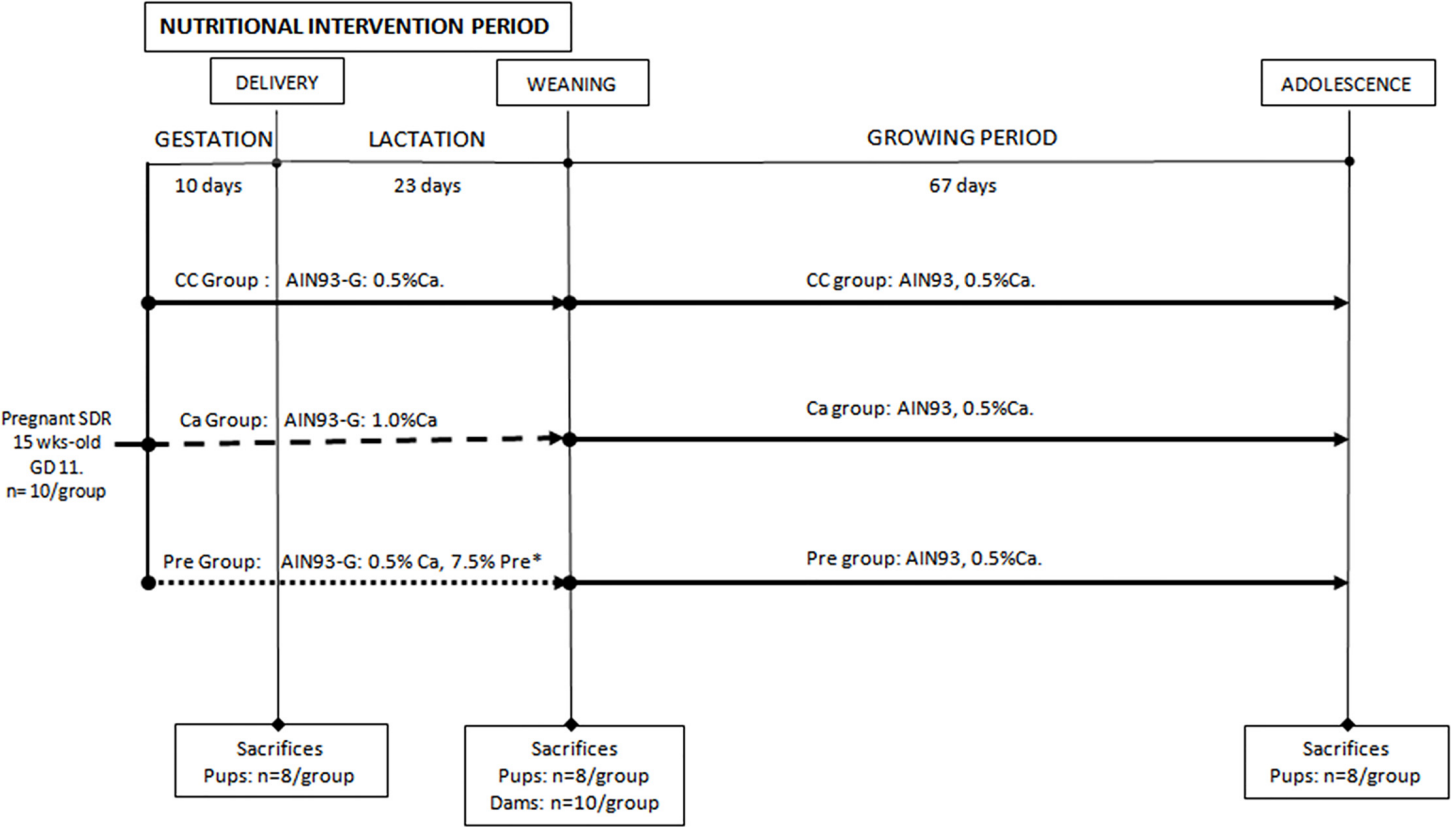
Experimental design. CC, Control group; Ca, Calcium-fortified group; Pre, Prebiotic oligofructose-enriched inulin supplemented group. Sacrifices were carried out at the end of each period, delivery, pups; weaning, pups and dams; adolescence, pups. *Prebiotic oligofructose-enriched inulin source provided by Orafti is a 1/1 mixture of oligofructose and high performance inulin.

All the rats were fed *ad libitum* and were given free access to de-ionized water during the entire period of pregnancy and lactation (Fig 1)

The compositions of experimental diets are defined in Table 1.

**Table 1:** Composition of the experimental diets used in the study.

	AIN93-G (CC group)	AIN93-G+Ca (Ca group)	AIN93-G+Pre (Pre group)
**Total Fat** (g/kg diet)	71.8	71.8	71.8
Soy oil	71.8	71.8	71.8
**Protein (g/kg diet)**	183.1	183.1	183.1
**Carbohydrate (g/kg diet)**	661.6	661.6	661.6
Cellulose	49.5	49.5	0.0
Prebiotic Oligofructose-enriched inulin*	0.0	0.0	75.0
**Minerals (/kg diet)**			
Calcium (g)	5.25	10.5	5.25
Magnesium (mg)	539.0	539.0	539.0
Manganesium (mg)	15.0	15.0	15.0
Phosphorous (g)	3.15	3.15	3.15
Zinc (mg)	39.9	39.9	39.9
**Vitamins (/kg diet)**			
Vitamin A (IU)	4200	4200	4200
Vitamin D_3_ (IU)	1200	1200	1200
Vitamin E (IU)	90	90	90
Vitamin K_1_ (mg)	1.08	1.08	1.08
**Energy** (kcal/kg diet)	3880.0	3880.0	3840.0

CC, Control group; Ca, Calcium-fortified group; Pre, Prebiotic oligofructose-enriched inulin supplemented group.

*Prebiotic oligofructose-enriched inulin source provided by Orafti is a 1/1 mixture of oligofructose and high performance inulin.

During these periods, control of dams’ weight and food intake was performed twice a week. After delivery, to standardize and minimize variation in the pups nutrition during suckling, litters from the same group were mixed, and 8 pups (5 females and 3 males) were randomly housed to each dam. Male pups were introduced to generate a litter as similar as possible to natural, avoiding any gender effect during the suckling, but were not subsequently used in the study. During their suckling period, the offspring received only the dam’s milk as source of feeding.

At weaning, female pups were separated from the dams and housed in groups of 4 per cage. Each animal received a controlled AIN93-G diet. Forty three days after delivery, the rats were individualized and fed with an AIN93-M maintenance purified diet [39, 40] until the end of their adolescence period (90 days).

Pup body weights were measured twice a week during the lactating period and weekly from weaning until the end of the adolescence for the offspring.

Before the sacrifices at the end of the weaning period for dams and of the adolescence, in the case of the offspring, urine was collected for 12 h in acidified tubes to measure biomarkers of bone resorption.

Sacrifices of dams and pups were carried out under fasting condition at the end of delivery (pups), their weaning period (23 days) (pups and dams), and at the end of their adolescence (90 days, pups). They were anesthetized via intraperitoneal with pentobarbital sodium, 30mg/kg of body weight (Abbott Laboratories, North Chicago, IL) and blood was sampled by cardiac puncture. Serum was isolated after centrifugation at 1,500 × *g* for 10 min at 4°C, frozen, and stored at -80°C until further analysis of bone biomarkers. Dams’ caecal content were collected, frozen and stored at -80°C for posterior analysis of pH. Femurs, tibiae and vertebrae, were also isolated and kept at -20°C until their analysis.

### Biochemical analyses

Serum osteocalcin was measured by a specific competitive enzyme-linked immunosorbent assay (ELISA) (Rat-MID^TM^ Osteocalcin EIA, IDS Inc., USA) and Alkaline phosphatase (AP) was measured by an automated colorimetric assay (Alcyon 300 analyzer; Abbott Laboratories, USA) using p-nitrophenyl phosphate as the substrate according to the International Federation of Clinical Chemistry and Laboratory Medicine[41]. Parathyroid hormone (PTH) was measured by a two-site ELISA (Rat intact PTH ELISA, ALPCO diagnostics, USA).

Biomarkers of bone resorption deoxypyridinoline (DPD) and pyridinoline (PYD) were measured in 12h-acidified urine, by using a competitive ELISAs (Metra tDPD and Microvue PYD, Quidel Corporation, USA). In order to normalize this parameter, urinary creatinine concentration was determined by the Jaffé method [42] using a clinical chemistry analyzer (Alcyon 300 analyzer, Abbott Laboratories, USA).

For the measure of the pH of caecal content, 0.5 g of the content was homogenized with 10 ml of deionized water (Milli-Q) and measured using a laboratory pHmeter (CRISON, Barcelona, Spain).

### *Ex-vivo* densitometry analysis

The whole BMD and BMC of total body at weaning and of isolated bones (femurs, tibiae and vertebrae) of dams and adolescent rats were measured by using peripheral dual-energy X-ray absorptiometry using a pDEXA^®^ densitometer (Norland Corp., Fort Atkinson, WI, USA). The femurs were measured from the femoral neck to the knee joint. The tibiae, including the fibula, were measured from the knee to the ankle joint and the vertebrae were measured from the inferior level of the lumbar vertebra 3 to the superior level of the lumbar vertebra 5 (LV3-LV5). In the current study, all measurements were performed by the same technician.

### Micro-CT analysis

Femurs, tibiae and/or vertebrae (LV4) were scanned *ex-vivo* at delivery (pups, femur and LV4), weaning (dams, femur, tibia and LV4, and pups, femur and LV4) and adolescence (pups, LV4) with high-resolution μCT using a Metris (XTek) Benchtop 160Xi CT scanner (University of Southampton) and a VivaCT-40 system (Scanco Medical AG, Brüttisellen, Switzerland). The femur and the tibia secondary spongiosa were scanned within the metaphysis below the growth plate. The LV4 spongiosa was scanned between the two growth plates. All scans from birth and weaning of the offspring bones were taken at 150kV, 60μA, using a molybdenum target. Samples at those ages were scanned at 19μm resolution with an exposure time of 534ms and 4x digital gain. Images in three-dimensional (3D) volume were reconstructed from the individual tiff images obtained from the CT scanner using the Metris (XTek) CT-Pro software. Cortical from LV4 at the end of adolescence was scanned at 70kV and 114μA, with a resolution of 20μm and an integration time of 800ms for the vertebrae samples. To obtain the 3D image of the LV4, a threshold 240 was used to binarize the cortex in this analysis. Threshold was adjusted in order to obtain the best 3D reconstructions.

Trabecular architecture parameters including trabecular number (Tb.N, measure of the average number of trabeculae per unit length, which is related with trabeculae density, in 1/mm), trabecular thickness (Tb.Th, the thickness of the trabeculae in mm), trabecular separation (Tb.Sp, the distance between trabeculae in mm), bone volume fraction (BV/TV, the ratio of spongy bone tissue including mineralized bone and osteoid), specific bone surface (BS/BV, to characterize the thickness and complexity of structures, in mm^-1^) and connectivity density (Conn.D, a measure of the degree of connectivity of trabeculae normalized by TV, in mm^-3^) were determined. Cortical parameters including volumetric BMD of the cortical bone (C.BMD), cortical thickness (C.Th, mean thickness of the cortical shell, in mm), and cortical porosity (C.Sp, defined as the volume of pores divided by the volume of cortical bone, in %) and the polar moment of inertia (pMOI, in mm^4^), as a structural index of resistance to torsion were also determined.

### Statistical analysis

Results were expressed as mean ± standard error of mean (SEM).

To evaluate differences attributable to the diet in the different markers, we performed a one-way ANOVA, followed by *post hoc* analysis with Fisher’s protected least significant difference mean separation test [43]. In groups which failed to exhibit normal distributions or equal variance, Kruskal-Wallis tests were performed. A value of p<0.05 was considered significant while a p-value between 0.05 and 0.10 was also noted as a trend. Statgraphics Centurion XVI (Stat Point Inc., Herndon, Virginia, USA) software has been used for data treatment and statistical analysis.

## Results

### Effect of maternal diets on dams’ and offspring weight

**Fig 2:**
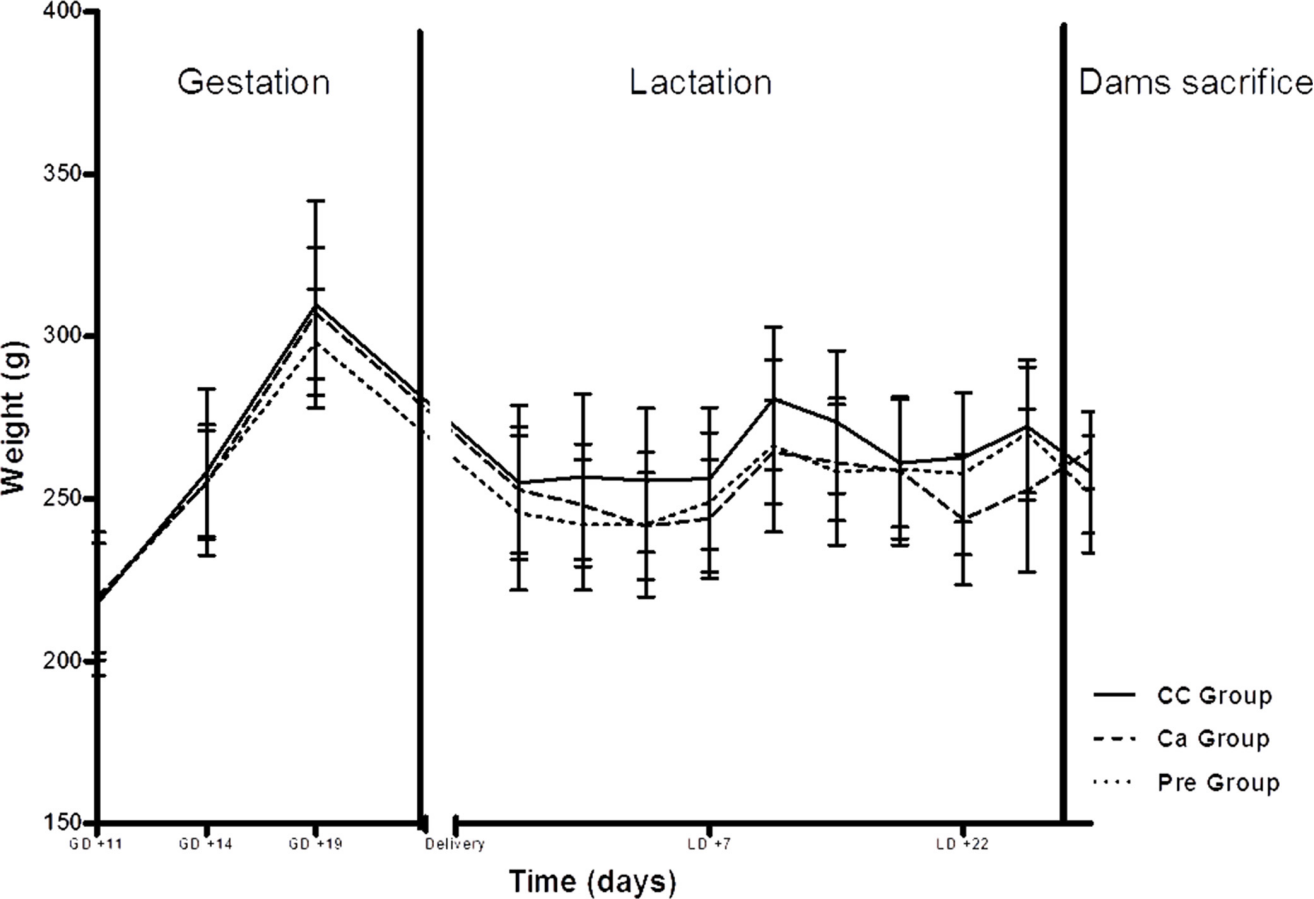
Maternal body weight evolution. Mean values with their standard deviations. (—)CC Group, Control group; (---) Ca Group, Calcium-fortified group; (···) Pre Group, Prebiotic oligofructose-enriched inulin supplemented group. GD, Gestational day; LD, Lactation day.

Dams’ weight evolution and food intake were similar between the CC and experimental groups during both gestation and lactation periods (Fig 2). At sacrifice no differences were found in dams’ weight (CC group: 258.0 ± 5.9g; Ca group: 265.0 ± 4.2g and Pre group: 251.4 ± 5.7g).

**Fig 3:**
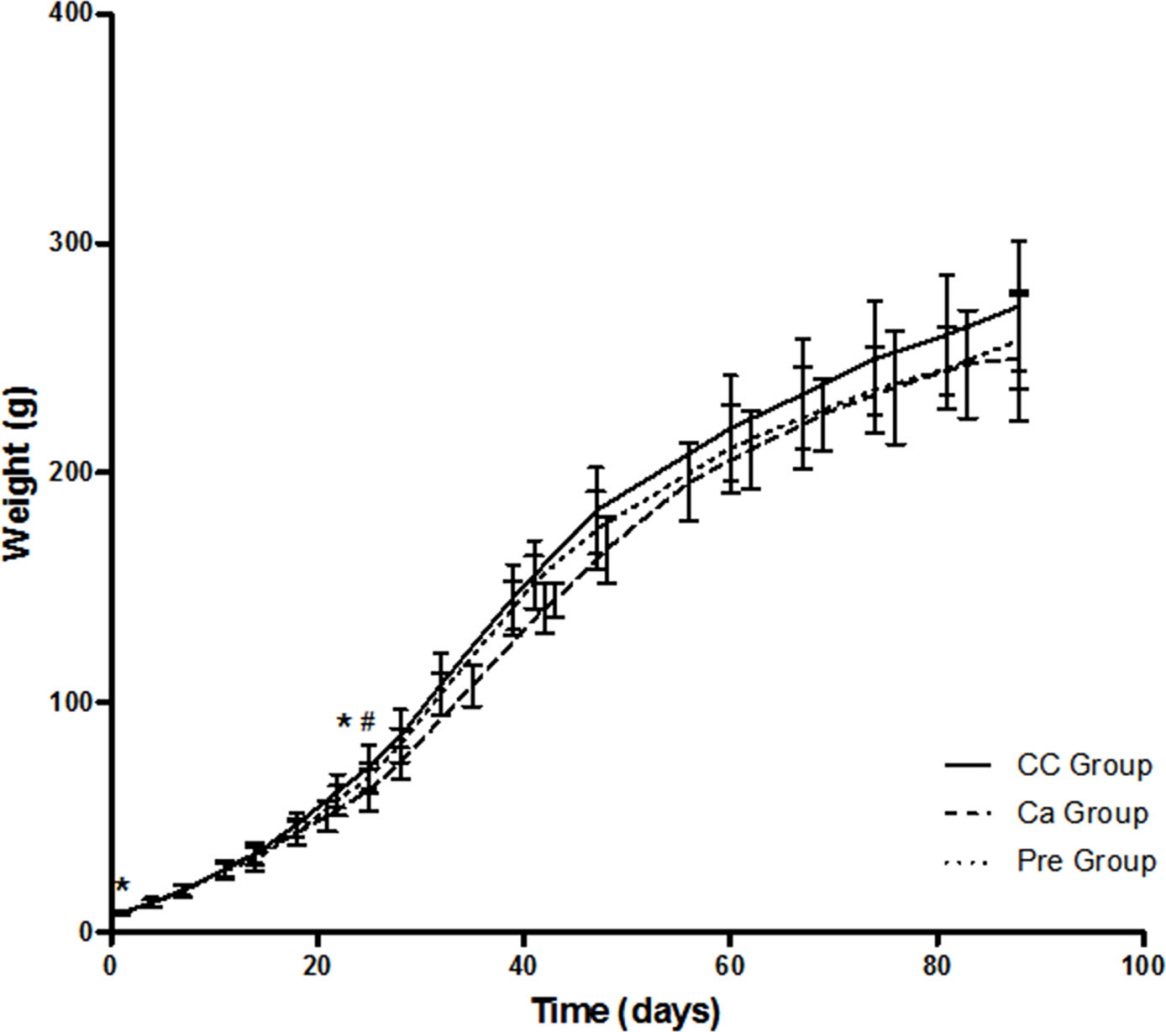
Pups body weight gain. Mean values with their standard deviations. (—)CC Group, Control group; (---) Ca Group, Calcium-fortified group; (···) Pre Group, Prebiotic oligofructose-enriched inulin supplemented group. Significant differences (p<0.05) * CC group *vs* Ca group; # Ca group *vs* Pre group.

Pups’ weight evolution was showed in Fig 3. Although nutrition intervention during gestation and lactation did not affect maternal body weight, the offspring body weight was significantly affected. At birth, the average weight of the offspring was significantly lower (*p =* 0.011) in the Ca group (8.24 ± 0.10g) compared to the average weight of the CC group (8.65 ± 0.12g). In contrast, no differences were found in pups weight between the Control and Pre groups (8.52 ± 0.13g).

At weaning, the pups from the Ca group mothers showed a significantly lower (*p*<0.05) body weight (62.0 ± 0.9g) compared to the CC group (72.2 ± 1.3g) and Pre group (67.6 ± 1.0g).

At the end of adolescence, offspring body weights were not different among the study groups (CC group: 272.7± 9.0g; Ca group: 250.0± 8.6g; Pre group: 257.7± 7.1g).

### Effect of maternal diets on dams’ bone health

#### Densitometry results

In absolute terms the femoral, the tibia and the vertebrae BMD and BMC were lower in dams which received the Ca-fortified diet with respect to the CC group, while these parameters were almost similar or even higher than control in dams fed with the diet supplemented with prebiotic oligofructose-enriched inulin (Table 2). Significant differences were only found on the tibia where CC and Pre groups showed a higher BMD (CC *vs* Ca, p = 0.049; Ca *vs* Pre, p = 0.009) and BMC (CC *vs* Ca, p = 0.029; Ca *vs* Pre, p = 0.026) with respect to Ca group.

**Table 2:** pDEXA analysis of bone mineral content (BMC) and density (BMD), from dams at sacrifice (end of lactation).

	CC group	Ca group	Pre group
	(n = 10)	(n = 10)	(n = 10)
**Femur**			
BMC (g)	0.3075 ± 0.0114	0.2813 ± 0.0128	0.3127 ± 0.0146
BMD (g/cm^2^)	0.1532 ± 0.0038	0.1419 ± 0.0044	0.1585 ± 0.0057
**Tibia**			
BMC (g)	0.2394 ± 0.0062	0.2122 ± 0.0094^CC^	0.2407 ± 0.0096^Ca^
BMD (g/cm^2^)	0.1229 ± 0.0019	0.1161 ± 0.0019^CC^	0.1258 ± 0.0032^Ca^
**LV3-5**			
BMC (g)	0.3064 ± 0.0106	0.2815 ± 0.0180	0.3138 ± 0.0165
BMD (g/cm^2^)	0.1473 ± 0.0029	0.1373 ± 0.0048	0.1500 ± 0.0039

Values are expressed as mean ± S.E.M. CC, Control group; Ca, Calcium-fortified group; Pre, Prebiotic oligofructose-enriched inulin supplemented group. Statistical significance was defined as *p*<0.05.

^CC^ significantly different *vs* the CC group

^Ca^ significantly different *vs* the Ca group.

#### Micro-structure results

**Fig 4:**
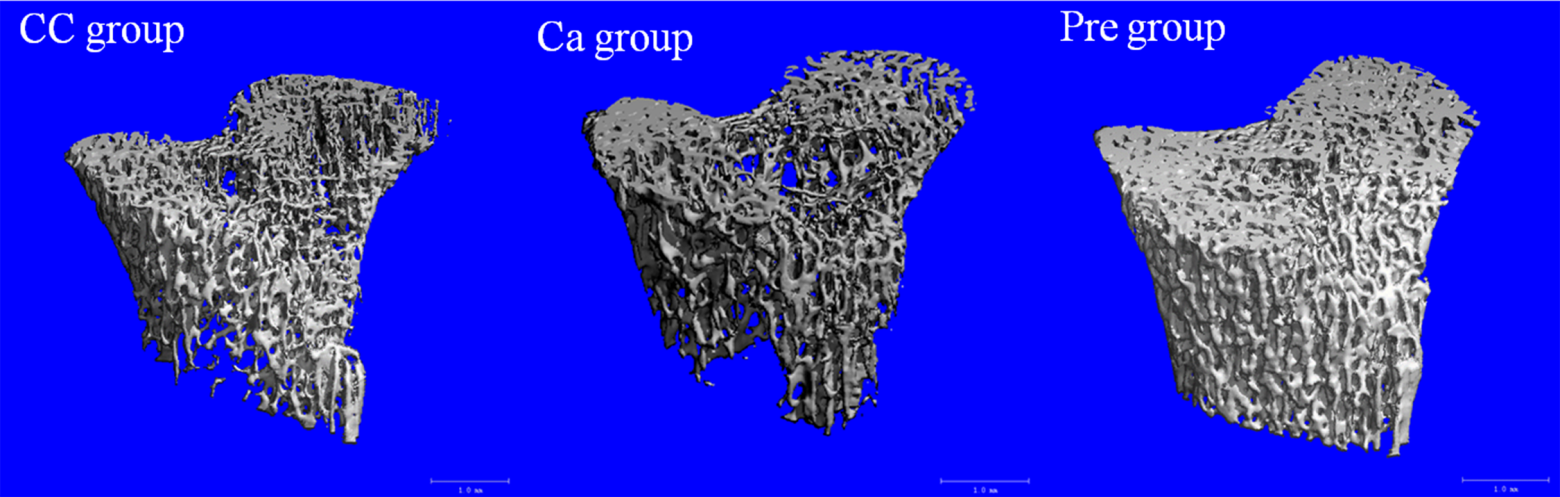
Representative micro CT images of trabecular bone in the tibia of dams. CC, Control group; Ca, Calcium-fortified group; Pre, Prebiotic oligofructose-enriched inulin supplemented group.

Micro-structure analysis of bones using micro CT technology pointed out the same pattern shown in pDEXA analysis. In general, and for all the bones analysed, the Pre group showed better trabecular micro-structure than the CC or the Ca groups (Table 3), being the tibia the most affected by maternal nutrition intervention (Fig 4). Thus, the tibia of dams in the Pre group showed a significant increase on trabecular thickness (p = 0.014) and a decrease in BS/BV ratio (p = 0.003) with respect to the CC group, that only reached a trend when comparing with Ca group (p = 0.073). This group also showed an increase of bone volume fraction and connectivity density with respect to CC (p = 0.033) and Ca groups (p = 0.017). Although no statistical differences were reached, it is also remarkable the higher trabecular number showed in dams from the Pre group (+15%) as compared to the CC group and +38% *vs* the Ca group and the lower trabecular space -6% *vs* the CC group and -23% *vs* the Ca group.

**Table 3:** Trabecular architecture of dams bones at sacrifice (end of lactation).

	CC group	Ca group	Pre group
	(n = 8)	(n = 8)	(n = 8)
**Femur**			
BV/TV	0.1584 ± 0.0116	0.1291 ± 0.0086	0.1521 ± 0.0154
BS/BV (1/mm)	34.29 ± 1.27	33.77 ± 0.62	32.59 ± 1.45
Tb. Th (mm)	0.0736 ± 0.0023	0.0715 ± 0.0018	0.0744 ± 0.0020
Tb. Sp (mm)	0.6185 ± 0.0482	0.7150 ± 0.050	0.6169 ± 0.0767
Tb. N (1/mm)	1.816 ± 0.1389	1.535 ± 0.0985	1.516 ± 0.077
Conn.D	70.01 ± 3.14	57.85 ± 4.25	73.97 ± 11.47
**Tibia**			
BV/TV	0.1274 ± 0.0094	0.1211 ± 0.0065	0.1723 ± 0.0213^CC^^,^^Ca^
BS/BV (1/mm)	43.55 ± 1.13	41.30 ± 0.85	38.33 ± 1.31^CC^
Tb. Th (mm)	0.0614 ± 0.0014	0.0631 ± 0.0011	0.0669 ±0.0017^CC^
Tb. Sp (mm)	0.4036 ± 0.0300	0.4956 ± 0.0364	0.3795 ± 0.0472
Tb. N (1/mm)	2.598 ± 0.1933	2.151 ± 0.1371	2.979 ± 0.3915
Conn.D	73.78 ± 4.57	66.18 ± 4.71	100.1 ± 14.58^CC^^,^^Ca^
**Vertebra (LV4)**			
BV/TV	0.2759 ± 0.0127	0.2580 ± 0.0143	0.2985 ± 0.0234
BS/BV (1/mm)	30.27 ± 1.01	31.83 ± 1.11	28.92 ± 1.18
Tb. Th (mm)	0.0750 ± 0.0018	0.0713 ± 0.0016	0.0768 ± 0.0019
Tb. Sp (mm)	0.2544 ± 0.0063	0.2571 ± 0.0095	0.2419 ± 0.0119
Tb. N (1/mm)	3.978 ± 0.081	3.947 ± 0.124	4.140 ± 0.179
Conn.D	104.0 ± 4.1	104.6 ± 3.6	99.1 ± 4.5

Values are expressed as mean ± S.E.M. CC, Control group; Ca, Calcium fortified group; Pre, Prebiotic oligofructose-enriched inulin supplemented group; BV/TV, Bone volume/total volume ratio; BS/BV, Bone surface/bone volume; Tb.Th, Trabecular Thickness; Tb.Sp, Trabecular Separation; Tb.N, Trabecular Number; Conn.D, Connectivity Density, LV4, lumbar vertebra 4. Statistical significance was defined as p < 0.05

^CC^ significantly different *vs* the CC group

^Ca^significantly different *vs* the Ca group.

#### Serum bio-markers

In general, no differences were found on serum biomarkers among the groups. Dams from the Ca group presented in the serum the higher concentration of markers associated to bone formation (osteocalcin and alkaline phosphatase) and of markers of bone resorption (DPD and PYD) (Table 4). Serum level of osteocalcin in the Ca group was statistically different with respect to the CC group (p = 0.007) but not respecting to the Pre group. Also the Pre group showed an increment with respect to the CC group for this parameter (p = 0.073).

**Table 4:** Bone biomarkers in serum and 12h-urine from the dams at sacrifice (end of lactation).

	CC group	Ca group	Pre group
	(n = 10)	(n = 10)	(n = 10)
**Bone formation biomarkers**			
Osteocalcin (mg/mol creatinine)	7489 ± 759.4	11133 ± 1008 ^CC^	9763 ± 869.6
Alkaline phosphatase (U/mmol creatinine)	3874 ± 726.3	5045 ± 639.8	3242 ± 380.3
**Bone resorption biomarkers**			
PTH (mg/mol creatinine)	1030 ± 113.0	880.5 ± 120.3	828.0 ± 90.74
DPD (μmol/mol creatinine)	166.3± 13.03	219.4 ± 26.59	187.5 ± 20.22
PYD (μmol/mol creatinine)	214.4 ± 11.46	242.4 ± 26.30	213.1 ±19.87

Values are expressed as mean ± S.E.M. CC, Control group; Ca, Calcium fortified group; Pre, Prebiotic oligofructose-enriched inulin supplemented group; Osteocalcin, Alkaline phosphatase and PTH (Parathyroid hormone) were measure in serum. DPD (Deoxypyridinoline) and PYD (Pyridinoline) were measured in 12h-acidified urine. Statistical significance was defined as *p* < 0.05.

^CC^ significantly different *vs* the CC group.

#### Caecal content pH

The pH of the caecal content from dams in the Pre group (8.083 ± 0.208) was significantly decreased respected to the CC group (8.872 ± 0.080; p = 0.001) and Ca group (8.558 ± 0.127; p = 0.035)

### Effect of maternal diets on offspring’s bone health and biomarkers

#### Results of offspring at delivery

At birth, densitometry analysis was not performed since the degree of bone mineralization of the new-born rat was not high enough and being below the detection limit of the densitometer. However, micro CT of trabecular parameters in femurs and the vertebrae were analysed based on the high sensitivity of this technique as compared to DEXA.

Pups from mothers in the Ca group (p = 0.004) and Pre group (p = 0.070) had lower femoral length in comparison to the CC group. Trabecular thickness was also lower in the Ca group (p = 0.001) and in the Pre group (p = 0.044) compared with CC group. On the other hand, pups in the Ca group presented a significant higher trabecular number (p = 0.000, *vs* CC and Pre) and BS/BV (p = 0.003, *vs* CC; p = 0.040 *vs* Pre) than those from mothers in the CC group and the Pre group (Table 5). Regarding trabecular structure of lumbar vertebrae, there was a positive increase in the trabecular number for both the Ca and Pre groups with respect to the CC group (+13% for the Ca group and +10% for the Pre group), although is this group which showed a higher trabecular thickness with respect to the other two groups (p = 0.019, *vs* CC; p = 0.045 *vs* Pre).

**Table 5:** Trabecular architecture of offspring bones at delivery period.

	CC group	Ca group	Pre group
	(n = 8)	(n = 8)	(n = 8)
**Femur**			
Length (mm)	4.283 ± 0.076	3.971 ± 0.058^CC^	4.099 ± 0.069
BV/TV	0.476 ± 0.030	0.468 ± 0.012	0.445 ± 0.033
BS/BV (1/mm)	19.47 ± 1.09	23.90 ± 1.23^CC^	22.41 ± 1.151^Ca^
Tb. Th (mm)	0.105 ± 0.006	0.079 ± 0.003^CC^	0.091 ± 0.005^CC^
Tb. Sp (mm)	0.117 ± 0.009	0.091 ± 0.004	0.118 ± 0.012
Tb. N (1/mm)	4.53 ± 0.13	5.89 ± 0.15^CC^	4.87 ± 0.22^Ca^
**Vertebra (LV4)**			
Length (mm)	0.739 ± 0.016	0.756 ± 0.020	0.693 ± 0.023
BV/TV	0.742 ± 0.029	0.671 ± 0.038	0.676 ± 0.034
BS/BV (1/mm)	17.39 ± 1.55	18.81 ± 0.71	21.12 ± 1.38
Tb. Th (mm)	0.122 ± 0.012	0.093 ± 0.003^CC^	0.098 ± 0.006^CC^
Tb. Sp (mm)	0.041 ± 0.005	0.040 ± 0.004	0.047 ± 0.005
Tb. N (1/mm)	6.35 ± 0.46	7.20 ± 0.37	6.97 ± 0.27

Values are expressed as mean ± S.E.M. CC, Control group; Ca, Calcium fortified group; Pre, Prebiotic oligofructose-enriched inulin supplemented group; BV/TV, Bone volume/total volume ratio; BS/BV, Bone surface/bone volume; Tb.Th, Trabecular Thickness; Tb.Sp, Trabecular Separation; Tb.N, Trabecular Number; LV4, lumbar vertebra 4. Statistical significance was defined as *p* < 0.05.

^CC^ significantly different *vs* the CC group

^Ca^significantly different *vs* the Ca group.

#### Results of offspring at weaning

No differences were found at this period in the whole body BMD and BMC of the female offspring (data not shown). However, in general terms, maternal nutritional intervention induced an improvement in the trabecular architecture of the vertebrae in the female offspring at weaning period. Although no significance was achieved, trabecular separation for the Ca group and the Pre group showed a decrease around -35% and -30% with respect to the CC group. In similar terms, a small increase with respect to the CC group was observed in the offspring from the Ca and Pre groups for BV/TV, by 7% and 5% respectively, and for trabecular thickness, by 11% for the Ca group and 7% for the Pre group. A significant decrease was found on BS/BV ratio (p = 0.033, *vs* CC; p = 0.012 *vs* Pre) and length (p = 0.002, *vs* CC; p = 0.005 *vs* Pre) in the Ca group *vs* both other groups (Table 6).

**Table 6:** Trabecular architecture of offspring bones at weaning period.

	CC group	Ca group	Pre group
	(n = 8)	(n = 8)	(n = 8)
**Femur**			
Length (mm)	19.44 ± 0.09	18.45 ± 0.28^CC^	18.81 ± 0.24
BV/TV	0.717 ± 0.033	0.657 ± 0.043	0.704 ± 0.008
BS/BV (1/mm)	6.548 ± 0.506	7.812 ± 0.929	7.467 ± 0.239
Tb. Th (mm)	0.312 ± 0.023	0.295 ± 0.020	0.269 ± 0.009
Tb. Sp (mm)	0.122 ± 0.012	0.154 ± 0.021	0.126 ± 0.011
Tb. N (1/mm)	2.318 ± 0.098	2.235 ± 0.071	2.539 ± 0.064^Ca^
**Vertebra (LV4)**			
Length (mm)	3.260 ± 0.098	2.737 ± 0.118^CC^	3.191 ± 0.048^Ca^
BV/TV	0.714 ± 0.038	0.764 ± 0.035	0.747 ± 0.038
BS/BV (1/mm)	8.068 ± 1.053	5.778 ± 0.267^CC^	8.418 ± 0.584^Ca^
Tb. Th (mm)	0.227 ± 0.042	0.252 ± 0.030	0.242 ± 0.016
Tb. Sp (mm)	0.112 ± 0.013	0.073 ± 0.006	0.081 ± 0.011
Tb. N (1/mm)	3.051 ± 0.288	3.150 ± 0.246	3.027 ± 0.052

Values are expressed as mean ± S.E.M. CC, Control group; Ca, Calcium fortified group; Pre, Prebiotic oligofructose-enriched inulin supplemented group; BV/TV, Bone volume/total volume ratio; BS/BV, Bone surface/bone volume; Tb.Th, Trabecular Thickness; Tb.Sp, Trabecular Separation; Tb.N, Trabecular Number; LV4, lumbar vertebra 4. Statistical significance was defined as *p* < 0.05

^CC^ significantly different *vs* the CC group

^Ca^significantly different *vs* the Ca group.

#### Results of offspring at adolescence

At the end of adolescence, pDEXA examination of appendicular bones, femur and tibia, did not differ among the nutritional intervention groups. In contrast, the lumbar vertebrae BMD of the Pre group is increased significantly compared to the CC (p = 0.039) and the Ca (p = 0.001) groups (Table 7), also BMC of this bone were influenced by maternal nutritional intervention, increasing in the Pre group more than 7% with respect to CC group and 16% with respect to Ca group. It is also remarkable the decrease of 8% that was found in the BMD of the Ca group with respect to CC group (p = 0.075). Based on those main differences on BMD and BMC of the lumbar vertebra, micro CT analysis was performed only in this bone at this period (Table 8).

**Table 7:** pDEXA analysis of the femur bone mineral content (BMC) and density (BMD) from offspring at adolescence.

	CC group	Ca group	Pre group
	(n = 8)	(n = 8)	(n = 8)
**Femur**			
BMC (g)	0.3643 ± 0.0090	0.3557 ± 0.0095	0.3580 ± 0.0164
BMD (g/cm^2^)	0.1782 ± 0.0033	0.1741 ± 0.0036	0.1787 ± 0.0042
**Tibia**			
BMC (g)	0.2770 ± 0.0079	0.2752 ± 0.0080	0.2697 ± 0.0106
BMD (g/cm^2^)	0.1376 ± 0.0026	0.1353 ± 0.0025	0.1368 ± 0.0027
**LV4-5**			
BMC (g)	0.3228 ± 0.0093	0.2982 ± 0.0144	0.3457 ± 0.0175
BMD (g/cm^2^)	0.1807 ± 0.0047	0.1670 ± 0.0043	0.1974 ± 0.0069^CC^ ^Ca^

Values are expressed as mean ± S.E.M. CC, Control group; Ca, Calcium fortified group; Pre, Prebiotic oligofructose-enriched inulin supplemented group; Statistical significance was defined as *p* < 0.05

^CC^ significantly different *vs* the CC group

^Ca^significantly different *vs* the Ca group.

**Table 8:** Trabecular and cortical architecture lumbar vertebra 4 from offspring at adolescence.

	CC group	Ca group	Pre group
	(n = 8)	(n = 8)	(n = 8)
**Trabecular Parameters**			
BV/TV	0.306 ± 0.007	0.373 ± 0.021^CC^	0.340 ± 0.017
BS/BV (1/mm)	28.370 ± 0.460	23.640 ± 0.723^CC^	25.440 ± 1.160^CC^
Tb. Th (mm)	0.071 ±0.001	0.082 ± 0.004^CC^	0.080 ± 0.003^CC^
Tb. Sp (mm)	0.161 ± 0.005	0.139 ± 0.007	0.156 ± 0.007
Tb. N (1/mm)	4.331 ± 0.103	4.524 ± 0.095	4.257 ± 0.115
**Cortical Parameters**			
C.BMD (mgHA/cm^3^)	385.40 ± 12.07	450.90 ± 14.95^CC^	435.20 ± 18.31^CC^
pMOI(mm^4^)	6.619 ± 0.352	7.026 ± 0.494	6.892 ± 0.355
C.Th (mm)	0.1674 ± 0.0045	0.1773 ± 0.0051	0.1836 ± 0.0058
C.Sp (%)	0.2743 ± 0.0086	0.2391 ± 0.0071^CC^	0.2578 ± 0.0106

Values are expressed as mean ± S.E.M. CC, Control group; Ca, Calcium fortified group; Pre, Prebiotic oligofructose-enriched inulin supplemented group; BV/TV, bone volume/total volume ratio; Tb.Th, Trabecular Thickness; Tb.Sp, Trabecular Separation; Tb.N, Trabecular Number; C.BMD Cortical Bone Mineral Density, pMOI, polar Moment of Inertia, C.Th, Cortical Thickness, C.Sp, Cortical Porosity. Statistical significance was defined as p < 0.05

^CC^ significantly different *vs* the CC group.

**Fig 5:**
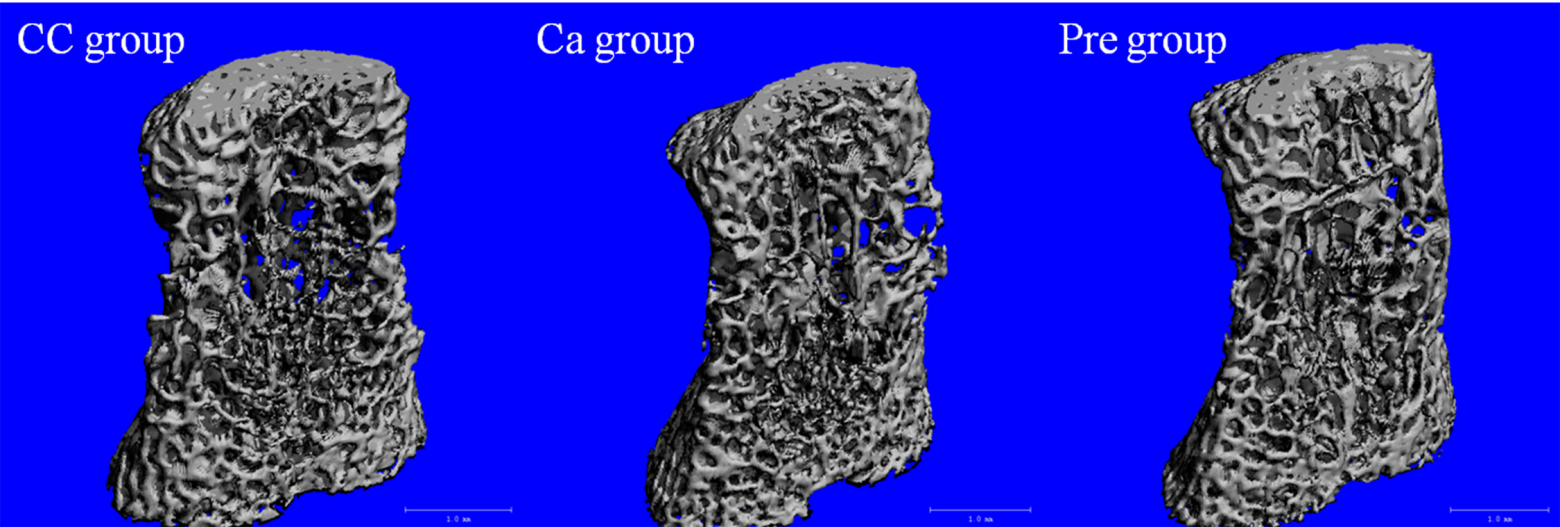
Representative micro CT images of trabecular bone in the LV4 of offspring at adolescence. CC, Control group; Ca, Calcium-fortified group; Pre, Prebiotic oligofructose-enriched inulin supplemented group.

The intervention on the mother, during gestation and lactation periods, with the Ca fortified or prebiotic oligofructose-enriched inulin supplemented diet produced a positive effect on the trabecular architecture of the vertebrae in their female offspring (Fig 5). BV/TV was significantly enhanced in the Ca group (22%) (p = 0.007) and also in the Pre group (11%), although no significant, in comparison to the CC group. Similar results were observed in trabecular thickness with an increase of 17% in the Ca group (p = 0.010) and 13% in the Pre group (p = 0.043) and in BS/BV ratio with a significant decrease of 17% (p = 0.001) and 10% (p = 0.021) respectively when compared with the CC group. The Ca group also showed a non-significant but higher decrease in the trabecular separation compared with the CC group (Table 8).

Cortical parameters of the vertebras of female offspring were also positively affected by the maternal nutritional intervention during the gestational and lactating periods (Table 8). The prebiotic oligofructose-enriched inulin supplementation produced an increase in C.BMD (13%) (p = 0.032 *vs* CC group) and in cortical thickness (9%), with a parallel reduction in cortical porosity (6%). Maternal Ca fortification also produced a significant increment in C.BMD (17%) (p = 0.006 *vs* CC group), with a significant decrease in the cortical porosity (13%) (p = 0.011 *vs* CC group), and a positive, but non-significant, increase of the cortical thickness (6%).

At the end of adolescence, higher concentrations of serum ALP was found in the Ca group with respect to the CC (p = 0.020) and Pre (p = 0.005) groups. Despite no significant differences were observed with respect to the CC group, a slight increase was also found in the bone formation marker, i.e. osteocalcin, and a decrease in bone resorption marker, i.e. PTH, for both Ca and Pre groups (Table 9).

**Table 9:** Bone biomarkers in serum and 12h-acidified urine from offspring at adolescence.

	CC group	Ca group	Pre group
	(n = 8)	(n = 8)	(n = 8)
**Bone formation biomarkers**			
Osteocalcin (mg/mol creatinine)	8404 ± 398	9698 ± 593	9323 ± 815
Alkaline phosphatase (U/mmol creatinine)	1475 ± 139	1962 ± 136^CC^	1311 ± 139^Ca^
**Bone resorption biomarkers**			
DPD (μmol/mol creatinine)	198.8 ± 16.5	210.2 ± 28.7	242.0 ± 19.8
PTH (μg/mol creatinine)	1313 ± 200	1019 ± 131	1107 ± 109

Values are expressed as mean ± S.E.M. CC, Control group; Ca, Calcium fortified group; Pre, Prebiotic oligofructose-enriched inulin supplemented group; Osteocalcin, Alkaline phosphatase and PTH (Parathyroid hormone) were measure in serum. DPD (Deoxypyridinoline) was measured in 12h-acidified urine. Statistical significance was defined as p < 0.05.

^CC^ significantly different *vs* the CC group

^Ca^significantly different *vs* the Ca group.

## Discussion

This study evaluated how maternal dietary intake of prebiotic oligofructose-enriched inulin supplementation or Ca fortification may exert a protective effect on maternal bone loss associated to gestation and lactation and may also affect the peak bone mass accretion and bone quality of the offspring during their development and growing periods.

Maternal skeleton plays a fundamental role in the offspring bone development, providing the calcium required for foetal development, principally during the third trimester of gestation, at delivery and for milk production during lactation. Thus, the maternal Ca storage is depleted over the course of pregnancy as the foetus accumulates 25 to 30g of Ca and during the nursing period approximately 210mg of Ca per day is transferred for maternal milk production [20, 44, 45].

In the current study performed in rats, results showed that a non-deficient maternal diet supplemented with prebiotic oligofructose-enriched inulin is able to prevent maternal skeleton from the transient osteopenia associated to pregnancy and lactation while Ca fortification in normal conditions did not exert any positive effect. Furthermore, a positive influence on the offspring bones during the very early stages of life was exerted by both ingredients.

Ca is an essential nutrient for pregnancy and lactation. Taking into account that most pregnant and lactating women do not consume the recommended quantity of Ca (1000-1200mg/day), supplementation is highly recommended to women in these periods. Thus, maternal supplementation with Ca has shown to have maternal and foetal skeletal benefits[46]; however it has also been related with other maternal health outcomes such as reduction of maternal risk of pregnancy-hypertension, preeclampsia and other complications [47]. It must be considered that most of the epidemiological studies have been done over deficient Ca population and, therefore, a supplementation with this mineral could mean an overall positive increase on bone health [20, 48]. However, not all human interventions have shown a positive effect of Ca supplementation during gestation and lactation on maternal bone health preservation, even in populations that consume very low Ca intakes [7]. Thus, in a recent publication, Jarjou *et al*. [49] have found that Ca supplementation of pregnant Gambia women with low Ca intake resulted in lower maternal bone mineral content during lactation.

In the present study, we found that dams from the Ca group showed the lowest BMD and BMC when compared with the CC and Pre groups. Moreover this group showed the most deteriorated micro-structure, denoting a greater de-calcification during the gestation and lactating periods. This means that, in our experimental conditions, no protection was obtained by Ca fortification on maternal bone health during these periods. In the same way, Shackelford *et al*. [50], in a study with well-nourished pregnant rats, observed a negative interaction between a Ca fortified diet during pregnancy and the minerals Fe, Zn, Mg and P levels on different tissues. Therefore, these results suggest that a unique supplementation with Ca during pregnancy and lactation could not be enough as it could generate an impairment on the metabolism of other essential minerals that are important for maternal health and for child development. In fact, some researchers have suggested that it may not just be Ca which promotes bone health but the synergistic effect of all of the nutrients in Ca rich foods [46, 51, 52].

New-born skeletal mineralization reflected the same effects of Ca fortification found in maternal bone. At delivery, the trabecular parameters in the Ca group indicated that the offspring had less mineralized spongy bones than the CC group. This finding could support that a maternal Ca-fortified diet in non-deficient conditions might have a negative effect on mineralization of the foetal skeleton by altering the absorption and metabolism of other key minerals such as Mg or P. Our data agrees with previous works reporting that high maternal Ca intake during pregnancy negatively affects foetus development. In the study of Schakelford *et al*. [50], a negative interaction was also observed between the Ca fortified diet during pregnancy and the foetal mineral tissue levels. In this study, foetuses from dams, fed with a Ca enriched diet, showed a decrease in the whole-body content Fe, Cu, P and Mg. Other studies have also showed that a high Ca supplementation in pregnant females can result in a negative effect on foetal bone mineral content [53, 54]. In contrast, an inadequate maternal store or intake of minerals could have adverse effects on the foetus, resulting in stillbirth, intrauterine growth retardation, and abnormal organ development [55].

At weaning, the Ca group offspring showed an improvement in the architecture of cancellous bone at lumbar vertebrae in comparison to the CC group, although it was not related to changes in the whole body BMD and BMC. This positive effect contrasts with the lower mineralization found at birth and confirms the significance of maternal Ca supplementation during lactation, i.e., period in which the main Ca source for the pup is breast milk. In this sense, expert committees recommended supplementation with Ca for premature infants fed with human milk. Furthermore, Lapillone *et al*. [56] concluded that healthy preterm infants fed with a Ca and P fortified formula, achieved a normal bone mass through an improvement in bone mineralization.

The most important differences were observed at the end of adolescence. The Ca group adolescent offspring presented a significant increase in the mineralization of the cancellous bone at lumbar vertebra compared to the CC group, as shown by micro CT analysis. The increase shown in trabecular thickness and the decrease in trabecular separation can be explained by an increased modelling rate on the trabeculae surface that produces denser cancellous bones in the Ca group offspring. In fact, this group also showed an increment in bone formation biomarkers, mainly in the alkaline phosphatase and a decrease, although no significant, in bone resorption biomarkers (PTH). In general, the lumbar vertebrae showed an improvement not only in cancellous bones but also in cortical bones. This improvement of the overall micro-structure of bones implies that the vertebrae of the Ca group offspring may have a greater resistance against bone breakage and collapse compared with the control group. These features were not confirmed using densitometric analysis, which failed to show any positive effect on BMD and BMC in the Ca group offspring compared with the CC group. Moreover, this was not in accordance with previous studies where maternal Ca fortification increased the amount of bone in offspring [54].

These results can be explained considering that our study was designed in non-deficient conditions, i.e. the amounts of Ca provided by the standard AIN-93 rodent diet were enough for covering adequate skeletal development. Similarly, other authors [3, 57, 58] reported that maternal Ca and vitamin D fortification did not influence clinical outcomes related to the offspring size at birth as well as the BMD and BMC in the progeny of women without apparent Ca and vitamin D deficiency. These results are in accordance with the concept of a threshold for Ca intake expressed by Matkovic and Heaney [59], i.e. intakes of Ca above the recommended level did not produce a further benefit.

On the other hand, prebiotic oligofructose-enriched inulin administration has showed to exert a more protective effect of dam’s bones against the transient osteopenia associated to pregnancy and lactation. Although no significant differences were found on BMD and BMC when comparing with the CC group, slightly higher values were obtained for all the analyzed bones. Taking into account that the cortical and the trabecular bone are included in the measurements of BMD and BMC, differences between groups could be dissipated by the effect of cortical bone that was less de-mineralized than the trabecular one. In fact, in humans, it is the trabecular bone the most affected during lactation, having a 3–10% decrease in trabecular BMC in women during 2 to 6 months of breastfeeding [60]. Similarly, Zeni *et al.[61]* have found that the contribution of the dam’s skeleton to the lactation period was greater in the areas with the highest trabecular composition. In this study, the proximal tibia of dams at weaning was the bone most affected by the de-mineralization during lactation, with a decrease in the BMD of 20% when compared to the non-pregnant control, while non-significant losses were found in the areas of cortical bone, such as distal and middle tibia.

Accordingly, our results showed that it is at microstructure level and in the cancellous bone, mainly of the tibia, where greater differences were found. The Pre group exhibited significant greater trabecular thickness, BV/TV and Conn.D and also a significantly lower bone surface-bone volume ratio than CC group denoting a higher protection, as trabeculae were lower de-calcified. In the same way, the cancellous bone in this group had lower trabecular space, greater trabecular number and significantly greater connectivity density than the Ca group denoting that dams from the Pre group presented higher bone strength and stiffness. In fact, bone strength depends not only on the quantity of bone tissue, evaluated by BMD using DEXA, but also on the quality, which is characterized by the three-dimensional organization of the trabeculae that is the microarchitecture[62].

Hence, prebiotic oligofructose-enriched inulin supplementation of the maternal diet during pregnancy and lactation achieved to protect maternal bone against loss produced by the maternal skeletal response to the demand of Ca necessary for foetal development. Although epidemiological studies observed no effect between bone mass loss during lactation and incidence of osteoporosis after menopause, it has been suggested that maternal BMD recovery after maternity fails to reach the pre-pregnancy BMD level [20], and, therefore, it is conceivable that a prevention of the osteopenia associated to lactation may exert a beneficial effect delaying and even decreasing the risk of osteoporosis later in life. Furthermore, it should also be taken into account the fact that the age at last pregnancy, that occurs increasingly later, approached the post-lactational recovery period to the perimenopause time, generating a situation in which the maternal bone has not been totally recovered between both periods. Therefore, a lower loss of bone during gestation and lactation could mean a greater protection against the osteoporosis associated to menopause.

In the same way, our results showed a beneficial effect of nutritional intervention in the mother during gestation and lactation over the offspring bone accrual. Prebiotic oligofructose-enriched inulin supplementation in the maternal diet did not show any outstanding effect on the bone mineralization or architecture in the offspring at delivery or at weaning. In contrast, this supplementation produced a significant increase in BMD and BMC in the lumbar vertebrae of adolescent offspring. The association between maternal prebiotic oligofructose-enriched inulin consumption and higher BMD/BMC in infants’ spines may be partially attributed to the increased mineral absorption from the maternal intestine, which could enhance the bio-availability and concentration of minerals in the placenta and breast milk. This increase might have a beneficial effect improving the ossification process in the foetus and the new-born but also for maternal bone against loss produced during gestation and lactation [63, 64]. Previous studies [15, 57] in healthy gestating women, observed an association between maternal mineral intake during the third trimester of pregnancy, and bone mass in their offspring at the age of 9 and 16. Both studies showed a positive relationship between mother Mg intakes, and the lumbar spine BMD in the infants. However, no such relationship was observed for maternal Ca intakes neither for the mother nor for the offspring [65]. In addition, bone health effects of inulin/scFOS are shown to be mediated by enhancing not only the intestinal absorption of Ca but also the absorption of other minerals with bone-sparing effects, such as Mg and P [66].

Moreover, prebiotic oligofructose-enriched inulin supplemented diet during gestation and lactation produces an increment on the structural features of the vertebra spongy bone. Compared to control pups, the offspring from the Pre group showed the same trabecular number but increased trabecular thickness (13%) and BV/TV (11%) that resulted in a denser bone, as shown by DEXA measurement (BMD). The Pre group offspring also showed a significantly higher C.BMD together with a greater cortical thickness in comparison to the CC group at the end of the adolescence. This denotes a greater periosteal apposition and probably a reduced endocortical resorption, thereby improving bone strength in the vertebrae. Cortical porosity was also reduced in this group (-6%) with respect to the CC group indicating a positive or well-balanced bone remodelling within each bone basic multicellular unit (BMU) that improved the toughness of the vertebra cortex. Furthermore, the Pre group showed a higher bone formation/bone resorption ratio (osteocalcin/PTH) with respect to CC group (8.42 *vs* 6.40), indicating an increase in the modelling rate that is reflected at the microstructure level. Altogether, these characteristics denoted a higher peak bone mass in the Pre group offspring, mainly promoted by the maternal prebiotic consumption during offspring early stages of life.

Another possible mechanism of action of prebiotics on the protection of the maternal skeleton and on the improved bone quality of the offspring would be through the production of short chain fatty acids (SCFA), such as butyrate, propionate, and acetate acids, by bacterial fermentation in the intestinal lumen. These acids have shown to influence bone health by different mechanisms. On the one hand, SCFA decrease the pH in the cecum, as is showed for the Pre group, dissolving insoluble Ca and Mg salts and accelerating their passive diffusion and absorption together with SCFA [26, 33, 67, 68]. On the other, SCFA have long term effects on skeletal development possibly involving the differentiation of bone marrow mesenchymal stem cells into osteoblasts [69] and stimulation of mineralized nodule formation[67, 70]. Nevertheless, further experiments are needed to explore the mechanisms of action of the programming effect of prebiotics.

## Conclusion

In conclusion, this study demonstrates for the first time that prebiotic oligofructose-enriched inulin supplementation during second half of gestation and lactation in rats exerted a protection on the trabecular structure of maternal bone against loss that occurs during pregnancy and lactating periods, especially in the tibia. This positive effect may lead to a greater protection against the bone loss that occurs later in life, when bones become thinner, resulting in a loss of strength to cracks and/or collapses. Furthermore, maternal prebiotic oligofructose-enriched inulin supplementation and Ca fortification produce a better structural base not only at spongiosa but also at the cortical vertebra of offspring bones, producing an increase in the offspring peak bone mass and, therefore, could help delaying the bone loss produced in adulthood and the risk of developing osteoporosis. Thus, based on our study results, prebiotic oligofructose-enriched inulin supplementation in non-deficient nutritional conditions may be considered as a plausible nutritional option for pregnant and lactating women rather than Ca supplementation, for maximizing the peak of bone mass in the offspring from the growing period till adolescence, and for protection of the maternal bone mass during gestation and lactation as a strategy to increase bone strength and to prevent bone fragility after menopause.
